# Warming-induced phenological mismatch between trees and shrubs explains high-elevation forest expansion

**DOI:** 10.1093/nsr/nwad182

**Published:** 2023-06-26

**Authors:** Xiaoxia Li, Eryuan Liang, J Julio Camarero, Sergio Rossi, Jingtian Zhang, Haifeng Zhu, Yongshuo H Fu, Jian Sun, Tao Wang, Shilong Piao, Josep Peñuelas

**Affiliations:** State Key Laboratory of Tibetan Plateau Earth System, Environment and Resources (TPESER), Institute of Tibetan Plateau Research, Chinese Academy of Sciences, Beijing 100101, China; Laboratoire sur les écosystèmes terrestres boréaux, Département des Sciences Fondamentales, Université du Québec à Chicoutimi, Chicoutimi G7H2B1, Canada; State Key Laboratory of Tibetan Plateau Earth System, Environment and Resources (TPESER), Institute of Tibetan Plateau Research, Chinese Academy of Sciences, Beijing 100101, China; Instituto Pirenaico de Ecología (IPE-CSIC), Zaragoza 50059, Spain; Laboratoire sur les écosystèmes terrestres boréaux, Département des Sciences Fondamentales, Université du Québec à Chicoutimi, Chicoutimi G7H2B1, Canada; State Key Laboratory of Tibetan Plateau Earth System, Environment and Resources (TPESER), Institute of Tibetan Plateau Research, Chinese Academy of Sciences, Beijing 100101, China; State Key Laboratory of Tibetan Plateau Earth System, Environment and Resources (TPESER), Institute of Tibetan Plateau Research, Chinese Academy of Sciences, Beijing 100101, China; College of Water Sciences, Beijing Normal University, Beijing 100875, China; State Key Laboratory of Tibetan Plateau Earth System, Environment and Resources (TPESER), Institute of Tibetan Plateau Research, Chinese Academy of Sciences, Beijing 100101, China; State Key Laboratory of Tibetan Plateau Earth System, Environment and Resources (TPESER), Institute of Tibetan Plateau Research, Chinese Academy of Sciences, Beijing 100101, China; State Key Laboratory of Tibetan Plateau Earth System, Environment and Resources (TPESER), Institute of Tibetan Plateau Research, Chinese Academy of Sciences, Beijing 100101, China; CREAF, Cerdanyola del Valles, Barcelona 08193, Spain; CSIC, Global Ecology Unit CREAF-CSIC-UAB, Bellaterra, Barcelona 08193, Spain

**Keywords:** chilling, climate warming, forcing, treeline shift, xylogenesis

## Abstract

Despite the importance of species interaction in modulating the range shifts of plants, little is known about the responses of coexisting life forms to a warmer climate. Here, we combine long-term monitoring of cambial phenology in sympatric trees and shrubs at two treelines of the Tibetan Plateau, with a meta-analysis of ring-width series from 344 shrubs and 575 trees paired across 11 alpine treelines in the Northern Hemisphere. Under a spring warming of +1°C, xylem resumption advances by 2–4 days in trees, but delays by 3–8 days in shrubs. The divergent phenological response to warming was due to shrubs being 3.2 times more sensitive than trees to chilling accumulation. Warmer winters increased the thermal requirement for cambial reactivation in shrubs, leading to a delayed response to warmer springs. Our meta-analysis confirmed such a mechanism across continental scales. The warming-induced phenological mismatch may give a competitive advantage to trees over shrubs, which would provide a new explanation for increasing alpine treeline shifts under the context of climate change.

## Teaser

Under warmer springs at the alpine treeline, growth reactivation is advanced in trees, but delayed in shrubs. This phenological mismatch creates a competitive edge for trees and offers a novel explanation for the dynamics of alpine treeline in the face of climate change.

## INTRODUCTION

Warming-induced range shifts are changing plant communities worldwide [1,2]. A major consequence of climate warming is the shift in the latitudinal and elevational ranges of plants, which has multiple implications for carbon and energy balances, hydrological processes and biodiversity [3–6]. Alpine treelines, the uppermost limit of tree growth forming the boundary between montane forest and alpine communities, are expected to advance upward in response to climate warming [7–9]. However, there is evidence that species interactions among woody plants play an important role in modulating range shifts along latitudinal [10,11] and at alpine treelines [12].

Species interactions, especially those between shrubs and trees, modify demographic processes, including tree recruitment, growth and mortality [1,12–15], thus affecting the final upward shift of alpine treelines. Experimental and observational studies show that shrubs could facilitate the germination of tree seeds and seedling establishment at the treeline by changing microclimate conditions, and increasing soil moisture, nutrients and diversity in symbionts, thus potentially promoting the upward migration of the alpine treeline with climate warming [13–16]. Shrub species have been found to act as nurseries or biotic bottlenecks for tree seedling survival and development at the treeline [12,13,17]. However, competition can also drive seedling establishment and treeline dynamics [12,18]. In fact, an index of shrub–tree competition on the Tibetan Plateau explained 70% of the variance in treeline dynamics over decadal scales [12]. Nevertheless, the ultimate mechanisms driving shrub–tree growth interactions in these harsh environments remain unknown, since facilitation related to tree recruitment may be transitory due to changes in abundance and cover of shrubs. Therefore, a question remains: will shrubs be able to outperform trees in the long term under a warmer climate? Answers to this question require analyzing long-term phenological and growth series, both at intra-annual and inter-annual scales, to capture the responses of woody plants to climate variability, and may allow a deeper understanding of how different life forms will respond to warmer conditions at high elevations.

Phenology is the phenotypic result of the interactions between genetics and environment. It has played an important role in the distribution range of woody plants, as well as in the survival and growth of plants, particularly in harsh environments such as alpine treelines [19]. Phenological differences between interacting species can mismatch the relative timings of life-history events, thereby changing competitive ability and distribution range [20]. The potential consequences of such a phenological mismatch could be amplified at the alpine treeline, where individuals grow under physiological stress due to limiting thermal conditions [19,21]. The phenological shifts between trees and shrubs could thus influence the long-term performance of these two growth forms by modifying their interactions, influencing the competition for resources and ultimately driving alpine treeline dynamics [12–14]. However, we still lack adequate and long-term information to assess how much climate warming will mismatch the phenology of interacting woody species at high elevations. The global pattern of growth–climate relationships of sympatric woody species in treeline ecotones also remains unclear [22–24]. A better understanding of the phenological events and growth patterns of alpine species is therefore needed for predicting how warming will affect the interactions between trees and shrubs.

Wood formation is the main process of growth and carbon sequestration of forest ecosystems [25]. The timings and dynamics of wood growth are crucial to assessing how trees respond to the climate and its changes [26,27]. Warm temperatures in spring promote an earlier onset of wood formation in trees [28,29]. However, few studies are available on the xylem phenology of other life forms, e.g. shrubs [30,31]. The lack of such phenological observations in sympatric shrub and tree species prevents us from disentangling the phenological mechanisms involved in species interactions at the alpine treeline.

At higher elevations, trees are more sensitive to temperature in terms of xylem growth than shrubs [22–24]; the former are more strongly coupled to atmospheric conditions, while the latter can benefit from being a smaller size and the warmer microclimate close to the ground [4]. Accordingly, we hypothesized that trees could benefit more than shrubs from warmer spring temperatures, resulting in an earlier growth reactivation compared to shrubs, and that such an asynchronous change in growth may mismatch the phenological events between trees and shrubs. In this study, first we tested these hypotheses using a unique, long-term series of xylem phenology in trees and shrubs at two alpine treelines of the Tibetan Plateau. In these long-term series we assessed the patterns and drivers of the cambial phenology of sympatric trees and shrubs in response to the temperature in spring, a critical season for wood formation [26–29]. Second, we performed a global meta-analysis combined with a process-based growth model to explore drivers of phenological growth shifts in response to warming between sympatric shrubs and trees at alpine treelines located in the Northern Hemisphere.

## RESULTS

### Phenological mismatch at the Tibetan Plateau

**Figure 1.**
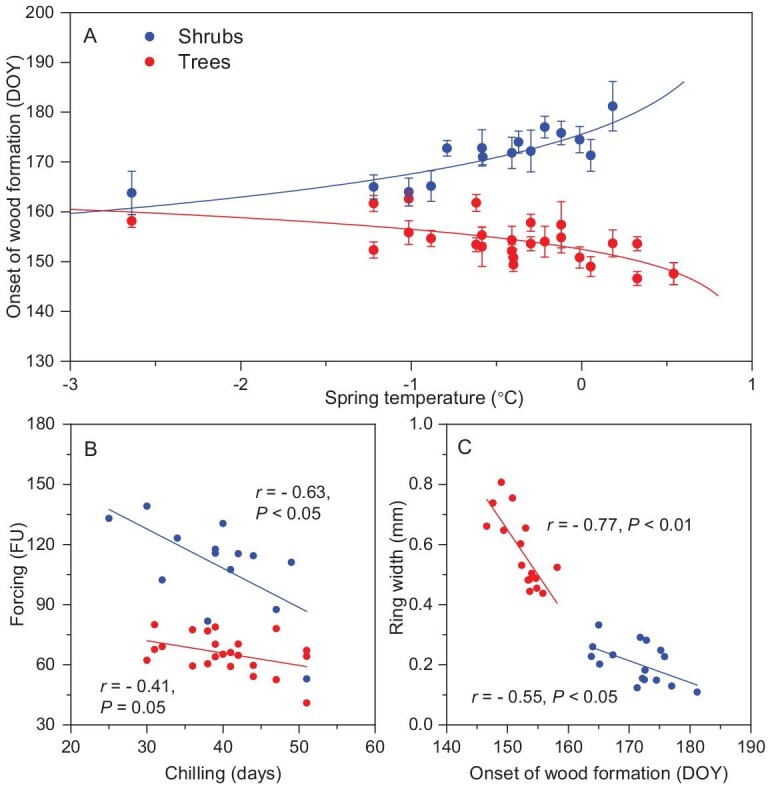
Response of the early cambial phenology of shrubs and trees to spring climate and its potential drivers at the two treelines on the southeastern Tibetan Plateau. (A) Effect of spring temperature on changes in cambial phenology for shrubs (blue points) and trees (red points; DOY, day of year). Values are means ± standard error (SE). Blue and red lines represent the dates of onset of wood formation predicted by linear models applying inverse transformations for shrubs and trees, respectively. (B) Relationship between chilling and forcing for the early cambial phenology of shrubs and trees. (C) Relationship between early cambial phenology and the ring width of shrubs and trees. The values of *r* and *P* were estimated using linear models.

The relationships between cambial phenology and spring temperature were non-linear in both shrubs and trees at the two treelines (Fig. 1A). After linearization of the trends, the regressions fitted the data adequately and produced reliable models (Supplementary Fig. S1), accounting for 69% and 40% of the variance in shrubs and trees, respectively (*P <* 0.001 in both cases). Significant differences were observed in the slope of the linear regressions between trees and shrubs (*t* = 7.65, *P* < 0.001, Supplementary Fig. S1 and Table 1), with divergent phenological responses to rising spring temperatures. Spring temperature and the date of onset of cambial phenology were positively correlated in shrubs, but negatively correlated in trees. For every additional Celsius degree in spring temperature, the onset of cambial phenology was delayed by 3–8 days in shrubs and advanced by 2–4 days in trees. An increase of 2°C in spring temperature would lead to a mismatch of ca. 20 days between trees and shrubs. A similar negative correlation was also observed between spring temperature and the date of bud swelling in tree saplings, with advancements of 6–8 days under a warming of +1°C in spring (Supplementary Fig. S2).

**Table 1.** Effects of chilling accumulation, life form (trees and shrubs) and interactions between chilling accumulation and life form on the heat requirement for the early cambial phenology at the two treelines on the southeastern Tibetan Plateau based on linear mixed models. Fixed effects were chilling and life form, whereas species and sites were regarded as random effects. Significance levels: *, *P* < 0.05; **, *P* < 0.01; ***, *P* < 0.001.

Model parameters	Estimate	SE	*t*
Intercept	186.47	20.21	9.23***
Chilling	−1.96	0.47	−4.21***
Life form	−92.50	27.08	−3.42**
Chilling × life form	1.28	0.63	2.05*

The heat requirement for the early cambial phenology was correlated negatively with the chilling accumulation in both shrubs and trees, with a significant negative correlation for shrubs (Fig. 1B). The chilling accumulation and its interaction with life forms have significant effects on heat requirement (Table 1). Shrubs were more sensitive to chilling accumulation than trees, as indicated by the slope of the regression, which was 3.2 times higher (*t* = 2.05, *P* < 0.05). Also, the onset of cambial phenology showed negative correlations with the final ring width for shrubs (*r* = –0.55, *P* < 0.01) and trees (*r* = –0.77, *P <* 0.05) (Fig. 1C), with linear regression having a higher slope in the latter (*t* = −3.26, *P* < 0.01, Supplementary Table 2).

### Phenological mismatch in the Northern Hemisphere

**Figure 2.**
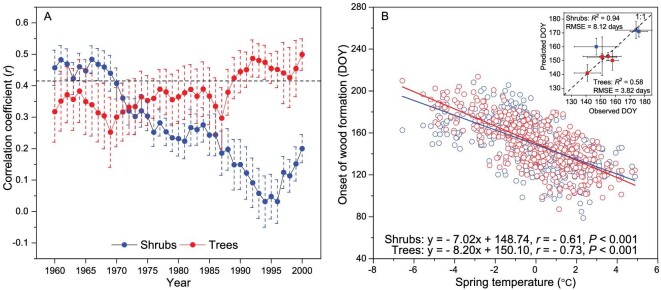
Response of onset and amount of xylem growth in treeline shrubs and trees to the temperature in the Northern Hemisphere. (A) Moving Pearson correlations (21-year-long intervals, 1960–2000 period) calculated between summer mean temperatures (June to August) and the ring-width chronologies for shrubs (blue points) and trees (red points). Values are means ± SE. The dashed line indicates the 0.05 significance level. (B) The relationship between onset of early cambial phenology predicted by the VS model and spring temperature indicates trees are more sensitive to spring warming than shrubs at alpine treelines. The small panel shows model performance indicated by the relationships between observed and simulated cambial phenology (day of cambial onset) at alpine treelines across mountainous areas located in the Northern Hemisphere (the dashed line is the 1 : 1 or x = y relationship). RMSE is the root mean square error.

We observed divergent patterns of growth–climate relationships between shrubs and trees at alpine treelines from 1960 to 2000 across the Northern Hemisphere (Fig. 2A). For trees, the correlation between summer (June to August) temperatures and tree-ring width has had a positive trend since the 1970s, becoming significant after the 1990s. However, significant and positive temperature–growth relationships were only observed before the 1970s in the case of shrubs.

A diverging pattern was also observed in the relationship between spring temperature and the onset of cambial phenology derived from the process-based growth model between 1960 and 2000 at alpine treelines in the Northern Hemisphere (Fig. 2B). Significant correlations (*P* < 0.01) were observed between the simulated and observed ring-width series for shrubs (mean ± SE, *r* = 0.44 ± 0.02) and trees (*r* = 0.58 ± 0.04) (Supplementary Fig. S3). The model was validated using *in situ* observations of the cambial phenology of shrubs and trees at alpine treelines across different mountainous areas of the Northern Hemisphere (Fig. 2B, and Supplementary Table 3). Spring temperature was negatively correlated with the simulated date of onset of cambial phenology in both trees (*r* = –0.73) and shrubs (*r* = –0.61, *P <* 0.001 in both cases) from 1960 to 2000 (Fig. 2B). However, shrubs were less sensitive to spring temperature than trees, as revealed by the lower slope of the linear regression (*t* = 2.04, *P* < 0.05, Supplementary Table 4).

The onset of simulated cambial phenology in shrubs and trees exhibited positive correlations with forcing, and negative correlations with chilling accumulation (Supplementary Fig. S4). Tree phenology had a stronger relationship with forcing (*r* = 0.30, *P* < 0.01) than chilling (*r* = –0.04, *P* > 0.05) accumulation. Shrub phenology was more related to chilling accumulation (*r* = –0.30, *P* < 0.01) than forcing (*r* = 0.24, *P* < 0.01). Compared with trees, shrubs exhibited a stronger correlation between simulated cambial phenology and winter temperature (Supplementary Fig. S4).

## DISCUSSION

We provide evidence that, under warmer spring conditions, cambial reactivation is advanced in trees but delayed in shrubs at the two treelines of the Tibetan Plateau. Our results are in line with phenological observations and previous studies on the cambial phenology of treeline conifers [26,28,29]. In particular, spring warming has advanced the onset of wood formation in conifers by a mean rate of ∼3 days per decade from 1960 to 2014 across the Tibetan Plateau [28]. The opposite phenological response to climate warming between trees and shrubs was also supported by our meta-analysis across the Northern Hemisphere, which revealed the divergent growth performance of sympatric woody species at the treelines, with trees outperforming shrubs in recent decades. Based on the cambial phenological records derived from the growth model, we further infer divergent effects of spring temperature on cambial phenology between trees and shrubs in the treelines of the Northern Hemisphere. Model outputs indicate that trees show a higher sensitivity to spring warming than shrubs, as expected. Our results show that, at higher elevations, shrubs are instead more influenced by chilling accumulation than trees.

Spring forcing and winter chilling are two main drivers determining spring phenology in plants [32–36]. Changes in these two factors have been widely used to explain the phenological changes of trees in response to increasing temperature [33–36]. A warmer spring advances growth reactivation in both primary meristems (buds and leaves) and secondary meristems (cambium) [28,35]. However, warmer conditions reduce chilling accumulation in autumn and winter, and delay spring phenology, mainly by increasing the heat requirement [33,36]. We quantified the effect of chilling accumulation on the heat requirement for stem-growth reactivation in trees and shrubs at the two treelines of the Tibetan Plateau. We demonstrated that, under the same amount of chilling, shrubs required more forcing accumulation than trees, leading to the delayed onset of cambial phenology. For trees, however, forcing was less dependent on chilling, as already demonstrated by previous studies [27,37], thus resulting in an earlier cambial phenology under a warmer spring. Based on the results of the process-based growth model, shrubs were more influenced by winter chilling at the alpine treelines across the Northern Hemisphere. The photoperiod may play a role in explaining divergent phenological shifts between trees and shrubs because different species have specific adaptations to the photoperiod [38]. However, experimental and observational evidence has revealed that trees tend to rely on temperature (spring warming and winter chilling) in regions with long and cold winters, whereas the photoperiod mainly regulates spring phenology in species growing at lower latitudes [39]. The divergent phenological response to warming between trees and shrubs therefore could be largely induced by different effects of chilling accumulation on the heat requirement for cambial reactivation between two woody species.

The timing of cambial phenology, especially its onset in spring, is one of the key drivers of xylem growth, not only for trees [26–29], but also for shrubs, as our study shows. In cold ecosystems, the length of the growing season is a determinant of radial growth, as indicated by tree rings, remote sensing [28,29,40] and our data (Supplementary Fig. S5). An earlier onset of cambial phenology leads to a higher number of cells produced by the meristems, which is positively correlated with the final ring growth of both trees and shrubs [30,41]. Therefore, an earlier growing season promotes wood growth in the Northern Hemisphere [28,29]. Given that cambial reactivation is influenced by spring temperatures, a warmer spring would cause an earlier onset of cambial phenology, resulting in increased cell production and xylem growth. Our study demonstrates that the diverging growth patterns observed between shrubs and trees at the treeline can be explained by the different directions of the shifts in cambial phenology in response to climate warming.

Warming-induced phenological mismatches have been widely documented across or within trophic levels [42–44], including those among sympatric plant species in deciduous forests [45]. Our findings demonstrate the occurrence of phenological mismatches at the elevation boundaries of woody vegetation. Mountains and high-latitude regions are experiencing warming rates more pronounced than lowland areas, especially during the winter [46,47]. Winter temperature has been forecasted to rise at a greater rate than spring temperature over the twenty-first century in mid-high latitudes of the Northern Hemisphere [48]. Such an asymmetric warming would further decrease the chilling accumulation and increase the heat requirement for reactivating growth in shrubs, potentially leading to delayed cambial reactivation, and consequent reductions in radial growth. In contrast, trees could benefit from an earlier growth reactivation under warmer spring conditions. An earlier growth reactivation might potentially increase the risk of frost damage in spring [49]. However, a cooling experiment demonstrated that late frost did not reduce xylem cell lignification in conifers at the alpine treeline [50]. Our findings suggest that climate change is mismatching phenology between trees and shrubs, potentially enlarging the gap in growth patterns between life forms at the alpine treeline.

**Figure 3.**
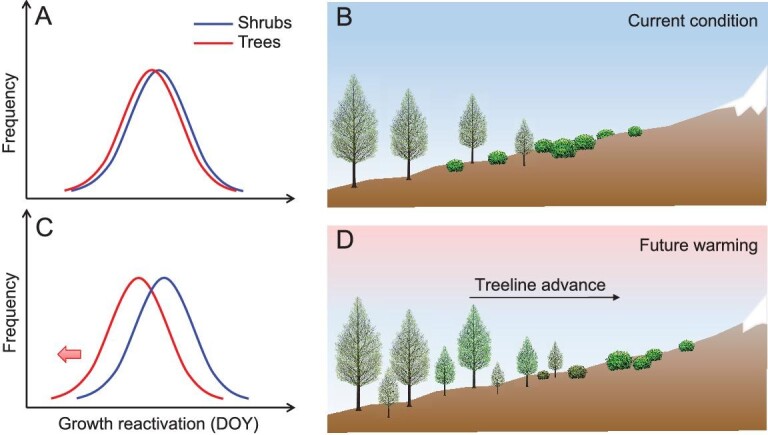
Early cambial phenological shift of shrubs (blue lines) and trees (red lines) and potential treeline shift under (A and B) current and (C and D) warmer climate conditions. Climate warming mismatches the early cambial phenology between these two life forms (A and C). An earlier cambial phenology may give a competitive advantage to trees over shrubs by increasing growth, carbon gain and improving resource availability, potentially promoting upward treeline shifts (B and D).

Our study paves the way for new ecological interpretations of treeline dynamics. Alpine treelines are strongly limited by low temperature and a short growing season [4]. Woody plants must modulate their primary and secondary growth during the short time window available to optimize carbon allocation to bud and woody tissues. Studies have revealed a temporal synchrony between bud and cambial phenology in conifers at both site and large spatial scales under a cold climate [51–53]. In the case of earlier growth reactivations of primary and secondary meristems, trees can benefit from a larger availability of resources, mainly light and soil nutrients, to enhance carbon assimilation [51,54]. This priority effect evidently facilitates not only mature trees, but also seedlings and saplings, because their growth rate and annual carbon assimilation depend mainly on the phenological events in spring [54]. With its long photoperiod and strong solar radiation, late spring and early summer provide the best conditions for the growth of treeline trees. An earlier spring phenology was associated with enhanced growth and carbon assimilation rates for tree seedlings, increasing their likelihood of survival [54]. Based on the decadal-scale correlation observed between tree recruitment and average spring temperature (Supplementary Fig. S6), a 1°C warming during spring on the Tibetan Plateau between 1960 and 2010 could potentially lead to a 50%–80% increase in the number of seedlings at the treeline. Our findings indicate that this warming effect can advance cambial reactivation by 2–4 days and bud swelling by 6–8 days. Given the relatively short growing season available at higher elevations [26], these changes represent a substantial time period. Regardless of whether shrubs and trees at alpine treelines interact through facilitation or competition, climate warming has the potential to drive divergent shifts in their phenology (Fig. 3A and C). This would involve a shortening of the growing season for shrubs and an extension of the growing season for trees. Consequently, the resulting phenological mismatch due to warming could confer a competitive advantage to trees over shrubs, enabling increased growth, carbon gain and improved resource availability. These factors may ultimately promote upward treeline shifts in cold mountainous regions (Fig. 3B and D).

## METHODS

### Study sites and species

Our study was carried out at the two alpine treelines in the Sygera Mountains, southeastern Tibetan Plateau, China. Two tree species, Blackseed juniper (*Juniperus saltuaria* Rehd. & Wilson) and Smith fir (*Abies georgei* var. *smithii* (Viguie & Gaussen) Cheng & Fu) and one shrub, *Rhododendron aganniphum* var. *schizopeplum* (Balf & Kingdon-Ward), were selected. Juniper grows on south-exposed slopes 4200 to 4400 m above sea level (a.s.l.). Fir is a shade-requiring conifer reaching the upper treeline at 4250 to 4400 m a.s.l., depending on the exposition. The low-stature vegetation is dominated by rhododendron. The study area is characterized by a cold and humid climate, with abundant rainfall during the monsoon season from June to September [12,55]. Snowpack at treelines usually persists from November until May with a maximum snow depth of ∼1 m.

We selected and studied two sites, SE-Site and N-Site, exposed at the southeast and north, respectively. At SE-Site (29°39.468′ N, 94°42.596′ E, 4390 m a.s.l.), 5–6 individual trees (juniper, fir) and rhododendron shrubs were selected in April 2007 and 2011, respectively. At N-Site (29°38.47′ N, 94°42.462′ E, 4380 m a.s.l.), 5–6 individual firs and rhododendron shrubs were selected in April 2012. The maximum heights of juniper and fir are 11.0 m and 13.5 m, respectively. Rhododendrons normally have a distinct main stem clearly differentiated from the branches, with a canopy ranging from 1.0 to 3.5 m in height, and a reproduction occurring mainly by seed. The characteristics of the sampled individuals are reported in Supplementary Table 5.

### Cambial phenology and temperature

The 10- and 8-year-long *in situ* observations of cambial phenology were assessed on a weekly time scale using histological sections of wood tissue collected in sympatric trees (juniper, fir) and shrubs (rhododendron) at two sites (see Supplementary Method 1). The periods studied for trees/shrubs were 2007–2016/2011–2017 at SE-Site and 2012–2017/2012–2019 at N-Site. The date of onset of cambial phenology, presented as the day of the year (DOY), was defined for each tree, year and site as the date of appearance of the first enlarging tracheids (juniper, fir) or vessels (rhododendron).

We collected air temperature with automatic weather stations (Campbell Scientific Corporation, Logan, UT) located at each site. Measurements were recorded at 3 m above ground every 30 minutes and stored in dataloggers. The daily and monthly means were calculated from the time series, and spring temperature was calculated for the period March–May.

### Growth–climate relationship of shrubs and trees at alpine treelines

We conducted a meta-analysis using published ring-width series to compare the responses of radial growth to temperature between sympatric shrubs and trees at alpine treelines located in the Northern Hemisphere. We focused on sites where temperature is the driving factor, and excluded sites strongly limited by other environmental factors, such as snow cover, wind or soil moisture. We selected studies according to the following criteria: (i) site location is situated at or near the alpine treeline, (ii) the correlation is higher between radial growth and summer (or growing season) temperature than other environmental factors, and (iii) the ring-width series are longer than 25 years and include the year 1990 to capture the recent warming trends. In total, we assembled a database from 11 alpine treeline sites in the Alps, Scandes, Sudetes, Tatras and Tibetan Plateau (Supplementary Table 6). Raw ring-width series were processed following standard dendrochronological methods (Supplementary Method 2). The series were standardized and detrended to obtain mean indexed standardized series (chronologies) which were used in successive analyses.

We used moving Pearson correlations (21-year-long intervals) to assess the changes in the relationships between summer temperatures (June to August) and ring-width chronologies from 1960 to 2000 for shrubs and trees, respectively. The June–August time window was set as the time span that covers most of the growing season in all regions [26,31]. We used monthly weather-station records for Tibetan Plateau sites and monthly E-OBS 26.0 gridded climate data (0.1° resolution) for sites located in Europe [56].

### Modeling the cambial phenology of shrubs and trees across alpine treelines

We applied the Vaganov-Shashkin process-based growth model [57] (hereafter VS model) to estimate the timings of the cambial phenology of shrubs and trees across alpine treelines from 1960 to 2000. The VS model has been widely used to simulate climate-driven variability in ring-width chronologies of trees and alpine shrubs [58,59]. It is able to simulate the onset of early cambial phenology by explicitly considering non-linear relationships between climate and growth [60]. A complete description of this model structure is given in Supplementary Method 3.

We calibrated the VS model by comparing simulated and observed standardized ring-width series (Supplementary Method 3 and Fig. S3). The genetic algorithm (GA) technique, a stochastic, population-based algorithm, was applied to estimate the optimum model parameters [61]. Initial values of these parameters were chosen from uniform distributions with appropriate ranges used in previous studies (Supplementary Table 7). We used GA to maximize the Pearson correlation (*r*) between the observed and simulated standardized ring-width chronologies, and retained the models with a significant correlation (*P* < 0.05). The key parameter settings (Supplementary Table 7), for example the minimum temperature for growth, were consistent with published observations of shrubs and trees at treelines [30,31,62].

The model was validated by comparing simulated and observed data of the cambial phenology of shrubs and trees at alpine treelines in the Northern Hemisphere (Supplementary Table 3). To the best of our knowledge, phenological data from the monitoring of most ring-width study sites do not exist. Therefore, the nearest available monitoring data at treelines were also collected from published literature [27,31]. There was good agreement between our modeled phenological series and data of *in situ* observations (Supplementary Table 3), suggesting that the model performance is robust. Finally, we simulated cambial phenology of both life forms involving 344 and 575 individual shrubs (6 species) and trees (5 species) from 11 alpine treeline sites (Supplementary Fig. S3 and Table S6).

### Assessment of temperature effects on the cambial phenology of trees and shrubs

We linearized the relationships between cambial phenology and spring temperature with the best type of x′ = log_10_(1 − x), and y′ = y, where x and y represent spring temperature and the onset dates of wood formation, respectively. We used linear mixed models (LMMs) to compare the relationships between cambial phenology and spring temperature. To link cambial phenology with bud phenology and tree establishments, we further assessed the relationship between spring temperature and bud swelling and tree regeneration in Smith firs at the same study sites (see details in Supplementary Method 4).

We calculated heat and chilling requirements for the onset of wood formation for shrubs and trees at each site. The heat requirement was calculated using a sigmoid function of the average daily air temperature [63] according to:


(1) \begin{eqnarray*}
{\mathrm{HU}} &=& \sum_{{t}_0}^{{t}_d} {{D}_{HU}} {\mathrm{if}}\,{T}_t > {T}_{th}\,\\
&&{\mathrm{where}}\,{D}_{HU}= \frac{{28.4}}{{1 + {{\mathrm{e}}}^{ - 0.185({{\mathrm{T}}}_t - 18.4)}}}
\end{eqnarray*}

where HU is the heat unit requirement for the onset of wood formation, *D_HU_* is the daily heat unit, *t_d_* is the date of onset for wood formation, *t*_0_ is the date of onset for heat accumulation (assumed here to be 1 January), *T_t_* is the mean daily air temperature and *T_th_* is the threshold temperature for heat accumulation. We used 0°C as the *T_th_*to calculate heat accumulation, considering that it was an optimal base temperature for most conifers [64].

Chilling temperature was calculated as the number of days in which the daily mean temperature ranged between −10 and 0°C, between −5 and 0°C, between 0 and 5°C and between −5 and 5°C, based on the period 1 September to 31 December of the previous year [65]. We used LMMs to assess the effect of forcing and chilling temperature on the early cambial phenology, considering both ‘site’ and ‘species’ as random factors. The Akaike Information Criterion (AIC) was used to rank models based on their parsimony and goodness of fit. Temperatures between −5 and 0°C were the most effective for the onset of wood formation for both shrubs and trees with the lowest AIC (Supplementary Table 8). Thus, we used this temperature range. Partial correlations were also used to assess the relationship between climate forcing (spring temperatures), chilling temperature (winter temperatures) and the modeled date of early cambial phenology based on VS model fits.

## Supplementary Material

nwad182_Supplemental_File

## Data Availability

The E-OBS 26.0e gridded database is available via https://www.ecad.eu/download/ensembles/download.php. The daily temperature data from weather stations were obtained from http://data.cma.cn/data/detail/dataCode/A.0012.0001. All observed data are included in the article and supplementary information. The code of the VS model written in MATLAB code is available at https://github.com/kanchukaitis/vsm. The detrending procedure for ring-width data was performed with the publicly available dplR package in R v.4.0.2.
