# Supplementary material for: Warming-induced phenological mismatch between trees and shrubs explains high-elevation forest expansion
Source: Natl Sci Rev. 2023 Jun 26;10(10):nwad182. doi: 10.1093/nsr/nwad182 (PMC10476895; doi:10.1093/nsr/nwad182)
Supplement: nwad182_Supplemental_File [file nwad182_supplemental_file.docx]

**Supplementary Data**

**Warming-induced phenological mismatch between trees and shrubs explains high-elevation forest expansion**

Xiaoxia Li, Eryuan Liang, J. Julio Camarero, Sergio Rossi, Jingtian Zhang, Haifeng Zhu, Yongshuo H. Fu, Jian Sun, Tao Wang, Shilong Piao, Josep Peñuelas

Eryuan Liang.

**Email:** liangey@itpcas.ac.cn.

**This PDF file includes:**

Supplementary methods 1-4

Supplementary figures 1-5

Supplementary tables 1-9

Supplementary references

Supplementary text

**Method 1. Sample collection and preparation**

Microcores of the stems were collected weekly from May until October at 1.0 - 1.3 m using a Trephor borer and fixed in a formalin-ethanol-acetic acid (FAA) solution^1^. In the laboratory, the microcores were dehydrated with successive immersions in ethanol and D-limonene, embedded in paraffin, and cut with a rotary microtome in transverse sections of 9 to 12 μm in thickness. The sections were stained with a mixture of safranin and astra blue, and then examined under a light microscopy using visible and polarized light.

The phases of xylem development included radial cell enlargement, secondary cell-wall thickening and lignification, and cell maturation^2^. Radially-enlarging tracheids and vessels contained a thin primary wall and had a radial diameter of at least twice that of cambial cells^3–4^. During wall thickening, the secondary cell walls showed birefringence under polarized light, which help to discriminate them from enlarging tracheids and vessels^2,5^. Finally, mature cells showed a wall completely developed and stained in red. The date of the onset of cambial phenology, presented as day of year (DOY), was defined for each tree, year, and site as the date of appearance of the first enlarging tracheids (juniper, fir) or vessels (rhododendron)^1,6^.

**Method 2. Chronology construction**

We used published ring-width series for shrubs and trees from 11 alpine treeline sites across the Northern Hemisphere covering the period 1960-2000, in whole or in part. Eleven series comprised raw ring-width data; the others were standardized ring-width chronologies (Supplementary Table 6). In total, we used ring-width data of shrubs (6 species) and trees (5 species) from 11 alpine treelines located in the Alps, Scandes and Tibetan Plateau.

For raw ring-width data, we removed biologically induced age/size trends by applying 67% cubic splines with a 1/2 cut-off time-series length. This detrending was done using the ‘dplR’ package^21^ in the R 4.0.5. package^22^. This method allows preserving annual to decadal scale variations in the detrended ring-width data. Each raw ring width was divided by the fitted value for each year. We built the standard site chronologies by using biweight, robust averages of individual detrended series to capture growth variability of individual sites and species. Other standardized chronologies were used for successive analyses.

We used moving Pearson correlations (21-year-long intervals) to assess the changes in the relationships between summer temperatures (June to August) and ring-width chronologies during 1960-2000 for shrub and trees, respectively. The June-August time window was set as the time span that covers most of the growing season in all regions^23^. We used monthly weather station records for Tibetan Plateau sites and monthly E-OBS 26.0 gridded climate data (0.1º resolution) for sites located in Europe^24^ .

**Method 3. Process-based growth modelling**

We used the Vaganov-Shashkin (VS) process-based model to simulate the early cambial phenology for shrubs and trees at alpine treelines across the Northern Hemisphere, based on the version implemented in MATLAB^25^. The VS model assumes that climatic influences are nonlinearly related to ring-width indices through controls on the rates of cambial activity processes. The model comprises an environmental and a cambial block. In the former block, the inputs are daily temperature, daily precipitation and site latitude (to estimate radiation). As an additional input, site ring-width series are also used as a target variable to maximize the coherence between the simulation and the target^26^. The daily growth is defined by the limitation of the growth rates dependent on daily temperature or soil moisture and weighted by the sunlight determined by the latitude of the ring-width study site^27^. Consequently, the daily growth rates Gt = min (gW, gT) gE, where gW, gT and gE are the daily partial growth rates based on soil moisture, temperature and solar radiation, respectively. The gE depends on the site latitude, as stated before, whereas gW and gT are nonlinear functions scaled between zero and one. Daily values of the integral growth rate are the final output of the environmental block and single input into the cambial block of the model. The cambial block converts it into the rate of radial growth for each cambial cell aligned along a radial cell file. Finally, the simulated ring-width chronology, as a key output of the cambial block, was transformed into a normalized number of differentiated xylem cells^26^. The simulated dates of the onset of the early cambial phenology (i.e., cambium division and cell radial enlargement) can be estimated as the occurrence of the first differentiating xylem cells. This model has a particular advantage in simulating the onset of early cambial phenology in both trees and shrubs and has been widely applied in conifer species^27–28^.

We assessed the robustness of the VS model simulations using the actual and predicted ring-width series for both life forms at alpine treelines across the Northern Hemisphere. The VS model contains 42 parameters in total, some of which have a significant impact on growth^26^. The genetic algorithm (GA) technique, a stochastic, population-based algorithm, was applied to estimate the optimum parameters^29^. Initial values of these parameters were chosen from uniform distributions with appropriate ranges used in previous studies (Supplementary Table 7). We used GA to maximize the Pearson correlation (*r*) between observed and simulated standardized ring-width chronologies, and retained the models with a significant correlation (*P* < 0.05). The model parameters were also calibrated considering available site information, such as soil moisture, root depth, temperature sum for initiation of growth. The key parameter settings (see Table 7), for example the minimum temperature for growth (T_1_), were consistent with published observations of shrubs and trees at treelines^6,30–31^.

Finally, we simulated phenological chronologies involving 337 and 575 individuals of shrubs (6 species) and trees (5 species), respectively, at 11 alpine treelines located across the Northern Hemisphere for the common 1960-2000 period (Supplementary Fig. S3 and Table 6). The model was verified by comparing the simulated and observations of early cambial phenology of both life forms at alpine treelines based on linear regressions and root mean square error (RMSE). To the best of our knowledge, the monitoring phenological data for most ring-width study sites does not exist. Therefore, the nearest available monitoring data at treelines were also collected from published literature (Supplementary Table 3). There was good agreement between our modeled phenological series and data of *in situ* observations (see details in Table 3), suggesting that the model performance is robust.

We used linear mixed models (LMM) to assess the effect of spring temperature, life form (trees and shrubs) and their interactions on the modeled date of early cambial phenology, considering both 'site' and 'species' as random effects. Partial correlations were also used to assess the relationship between forcing (spring temperature), chilling temperature (winter temperature) and date of the early cambial phenology based on VS models. Climatic data used in this model (period 1950-2020) was obtained from the nearest meteorological station to the sites on the Tibetan Plateau and from the E-OBS 26.0 gridded database with a resolution of 0.1º for sites located in Europe^24^ (Supplementary Table 9). We corrected the daily temperature data using linear regressions^34^ for the two treeline sites situated in the Sygera Mountains (SYS and SYN), and a lapse rate of 0.60 ºC / 100 m for other sites by calculating the altitudinal differences between the sample site and the station site or the center of the E-OBS grid.

**Method 4. Relationship between spring temperature and bud phenology/tree regeneration in Smith fir**

Bud phenology of Smith fir (*Abies georgei* var. *smithii*) was monitored at four sites along an altitudinal gradient on a north-facing slope in the Sygera Mountains, southeastern Tibetan Plateau. At each site, 10 saplings of Smith fir were randomly selected for taking successive observations. Saplings ranged from 0.39 to 1.83 m in height, and were 10 to 44 years old. Their phenology was weekly recorded between April and October during 2013–2019. The onset of bud swelling was defined as the date when over half of the trees started growing (i.e., when the new terminal bud is clearly visible) at each site^35-36^. Temperature data was recorded with an interval of 30-min using a temperature logger (TidbiT v2 Temp UTBI-001, Onset Computer Corporation, Bourne, MA), installed in each plot 1–2 m above ground under the canopy of a mature tree near the saplings. Pearson correlation analysis was used to assess the relationship between spring temperature (March-May) and date of bud swelling.

Tree regeneration data of Smith fir treeline in the same study site was collected from published literature^37^. This data was calculated based on reconstructed age structures of tree populations during 1960-2010 from three Smith fir treeline plots (30 ×150 m) located in the Sygera Mountains, south-eastern Tibetan Plateau. We extended the time series of air temperatures at the treeline back to 1960 based on the close relationship in the daily temperature variation between Nyingchi weather station nearby and our treeline automatic weather stations (*r* = 0.89, 2007-2010). Since tree recruitment may show lagged responses to temperature changes, we correlated average spring temperature and tree regeneration at decadal scales according to previous studies^38^.

**Figures 1-5:**


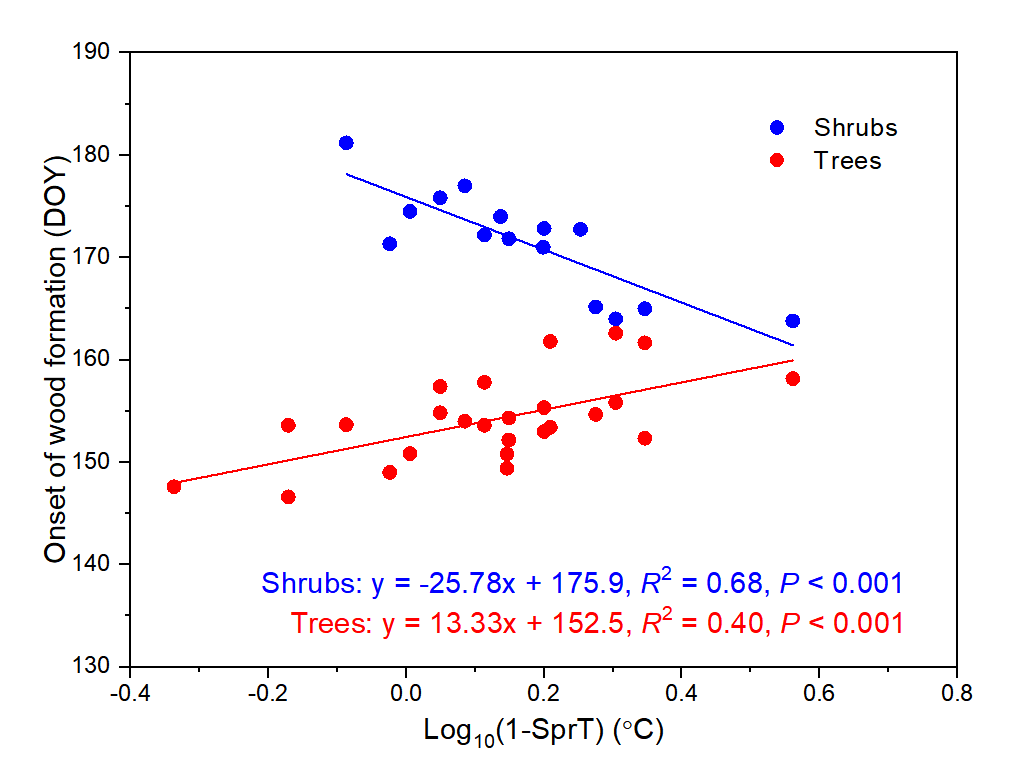


**Fig. S1** Relationship between spring temperature after inverse transformations (Log _10_ (1-SprT)) and the early cambial phenology (onset of wood formation, DOY) of trees (red points and line) and shrubs (blue points and line) at two treelines on the southeastern Tibetan Plateau.

**Fig. S2** Relationship between spring temperature and date of bud swelling (DOY) of 40 Smith fir saplings during 2013-2019 along an altitudinal gradient in the Sygera Mountains, south-eastern Tibetan Plateau.

**Fig. S3** Comparison between the modeled (red lines) and observed (black lines) indexed ring width series of shrubs and trees over their common periods at alpine treelines located across the Northern Hemisphere. The significant (*P* < 0.05) and positive correlations between the modeled indexed ring width series confirm the model performance is robust. Statistics show the Pearson correlation coefficient (*r*) and its significance levels (*P*).


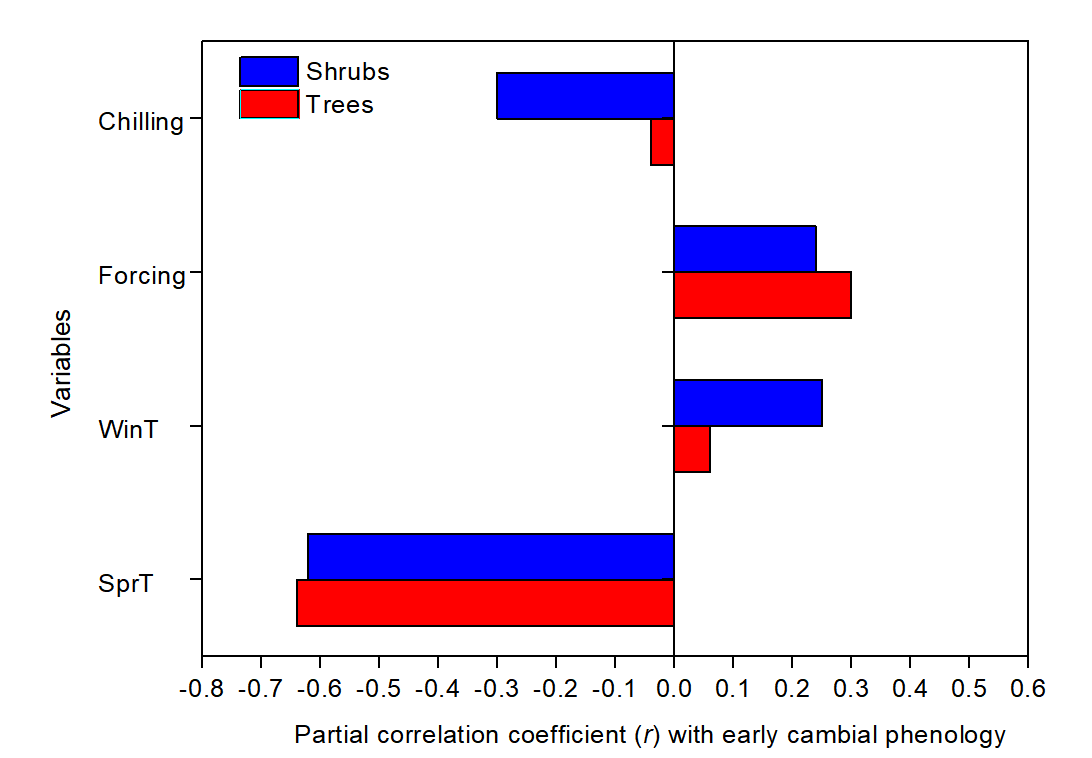


**Fig. S4** Partial correlations calculated between the early cambial phenology and winter chilling, spring forcing and winter (WinT) and spring (SprT) temperatures in shrubs (blue bars) and trees (red bars) at alpine treelines located across the Northern Hemisphere. Correlations were highly significant (*P* < 0.001) in all cases.

**Fig. S5** Relationships between the duration of xylem growth, growth rate and final xylem growth amount of trees (red symbols and lines) and shrubs (blue symbols and lines) during 2007-2010 and 2012-2015, respectively, at two treelines located in the Sygera Mountains, south-eastern Tibetan Plateau.


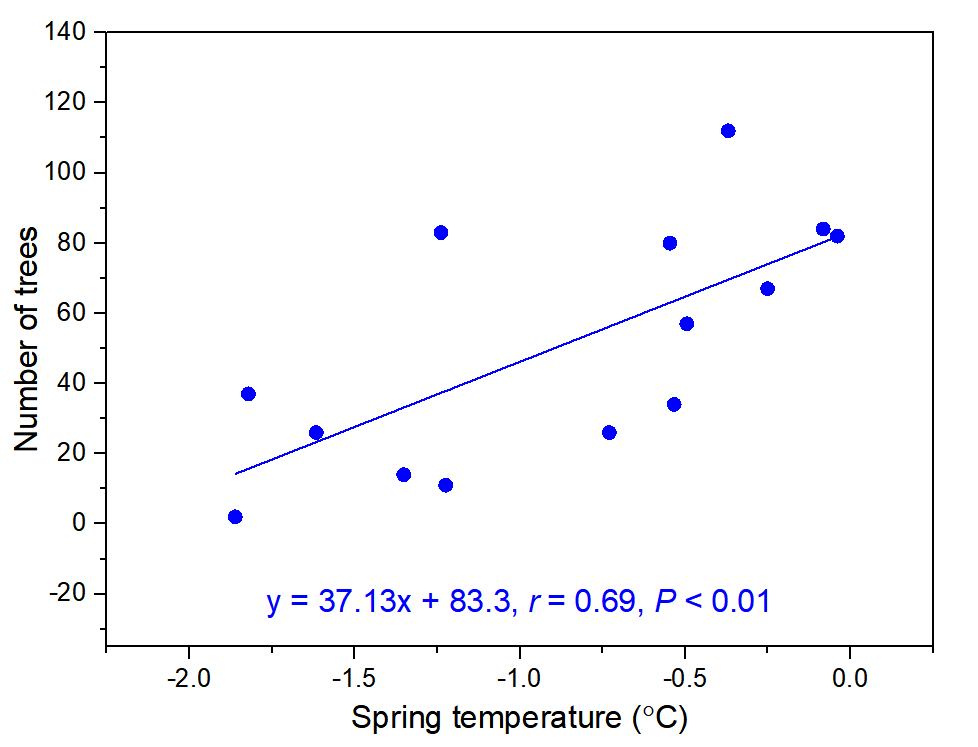


**Fig. S6** Relationship between average spring temperature and decadal tree regeneration of Smith fir at treeline during 1960-2010 in the Sygera Mountains, south-eastern Tibetan Plateau.

**Tables 1-9:**

**Table 1**. Effects of spring temperature after inverse transformations (Log _10_ SprT), life form (trees and shrubs) and their interactions on the early cambial phenology at the two treelines on the southeastern Tibetan Plateau. Fixed effects were Log _10_ SprT and life form, whereas species and sites were regarded as random effects. Abbreviations: SE, standard error; Significance levels: **, *P* < 0.01; ***, *P* < 0.001.

| Model parameters | **Estimate** | **SE** | ***t*** |
| --- | --- | --- | --- |
| Intercept | 175.91 | 2.93 | 60.06** |
| Log**_10_** SprT | -25.78 | 4.44 | -5.81*** |
| Life form | -23.03 | 3.57 | -6.46 |
| Log**_10_** SprT × life form | 39.39 | 5.15 | 7.65*** |

**Table 2.** Effects of early cambial phenology (onset of wood formation), life form (trees vs. shrubs) and their interactions on the final ring width at the two treelines located on the southeastern Tibetan Plateau. Fixed effects were early cambial phenology and life form, whereas sites were regarded as random effects. SE, standard error. Significance levels: *, *P* < 0.05; **, *P* < 0.01.

| Model parameters | **Estimate** | **SE** | ***t*** |
| --- | --- | --- | --- |
| Intercept | 1.47 | 0.642 | 2.28* |
| Onset | -0.01 | 0.003 | -1.97 |
| Life form | 3.72 | 1.391 | 3.35****** |
| Onset × life form | -0.02 | 0.007 | -3.26****** |

**Table 3.** Detailed information of monitoring cambial phenology of shrubs and trees at alpine treeline sites located across the Northern Hemisphere used for the verification of the VS model. DOYs are means ± SE. See more site information in **Table 6**.

| Site code | Modeled date (DOY) | Monitored date  (DOY) | Monitored years | Life form | Species | Latitude (N) | Longitude (E) | Elevation (m a.s.l.) | No. individuals | Ref**.** |
| --- | --- | --- | --- | --- | --- | --- | --- | --- | --- | --- |
| KRK | 147 ± 15 | 160 ± 6 | 2016-2017 | Shrub | *Pinus mugo* | 50.72 | 15.67 | 1370 | 6 | 31 |
| KRK | 158 ± 4 | 150 ± 7 | 2016-2017 | Tree | *Picea abies* | 50.72 | 15.67 | 1370 | 6 | 31 |
| CS | 141 ± 9 | 141 ± 6 | 2003-2004 | Tree | *Picea abies* | 45.05 | 6.66 | 2030 | 5 | 32 |
| DL | 151 ± 13 | 153 ± 14 | 2009-2012,2014 | Tree | *Juniperus przewalskii* | 36.00 | 98.18 | 4210 | 5 | 33 |
| SYS | 176 ± 6 | 171 ±4 | 2011-2017 | Shrub | *Rhododendron*  *aganniphum* | 29.66 | 94.71 | 4390 | 5-6 | Our data |
| SYN | 174 ± 14 | 172 ± 6 | 2012-2019 | Shrub | *Rhododendron*  *aganniphum* | 29.64 | 94.70 | 4380 | 5-6 | Our data |
| SYS | 151 ± 10 | 152 ± 3 | 2007-2016 | Tree | Abies georgei | 29.66 | 94.71 | 4390 | 5-6 | Our data |
| SYN | 155 ± 6 | 153 ± 3 | 2012-2017 | Tree | Abies georgei | 29.64 | 94.70 | 4380 | 5-6 | Our data |

**Table 4.** Effects of spring temperature (SprT), life form (trees vs. shrubs) and their interactions on the early cambial phenology based on VS models at alpine treelines across the Northern Hemisphere according to linear mixed models. Fixed effects were spring temperature and life form, whereas species and sites were regarded as random effects. SE, standard error. Significance levels: *, *P* < 0.05; ***, *P* < 0.001.

| Model parameters | **Estimate** | **SE** | ***t*** |
| --- | --- | --- | --- |
| Intercept | 149.44 | 0.592 | 254.22*** |
| SprT | -7.64 | 0.29 | -26.72******* |
| Life form | -0.72 | 0.59 | -1.22 |
| SprT × life form | 0.58 | 0.29 | 2.04* |

**Table 5.** Basic characteristics of sampled trees and shrubs at two treeline sites located on south-eastern Tibetan Plateau, including age, diameter at breast height (DBH, measured at 1.3 m) and height (H). Values are means ± SD.

| Sites | Species | Age (years) | DBH (cm) | H (m) |
| --- | --- | --- | --- | --- |
| SE-Site | *Juniperus saltuaria* | 252 ± 20 | 32.0 ± 3.3 | 9.4 ± 1.2 |
|  | *Abies georgei* var. *smithii* | 201 ± 24 | 34.4 ± 5.0 | 11.9 ± 0.9 |
|  | *Rhododendron aganniphum* | 204 ± 39 | 8.2 ± 1.5 | 3.2 ± 0.4 |
| N-Site | *Abies georgei* var. *smithii* | 246 ± 12 | 42.3 ± 5.8 | 12.3 ± 1.1 |
|  | *Rhododendron aganniphum* | 161 ± 20 | 6.4 ± 1.6 | 2.8 ± 0.5 |

**Table 6.** Detailed information on the ring-width data used in the study at alpine treeline sites across the Northern Hemisphere. Sites with climate data obtained from nearby weather stations are indicated by asterisks (*). Raw ring-with data are indicated by asterisks (*) and other statistics correspond to standardized ring-width chronologies.

| **Code** | **Site** | **Species (shrubs/trees)** | **Shrub information** | | **Latitude (N)** | **Longitude (-W, +E)** | **Elevation (m a.s.l.)** | **No. shrubs/trees** | **Chronology length (shrubs/trees)** | **Refs.** |
| --- | --- | --- | --- | --- | --- | --- | --- | --- | --- | --- |
|  |  |  | **Leaf habit** | **Maximum height (cm)** |  |  |  |  |  |  |
| VOL | Central Norwegian Scandes,Vole | *Empetrum hermaphroditum* / Betula pubescens | Evergreen | 30 | 61.90 | 9.14 | 1100 | 20/14 | 1934-2013/  1925-2003 | 7–8 |
| JES | Central European, Jes | *Pinus mugo* / Picea abies | Evergreen | 200 | 50.24 | 16.89 | 1410 | 32/55 | 1919-2006*/  1919-2006* | 9 |
| TAT | Central European,Tat | *Pinus mugo* / Picea abies | Evergreen | 200 | 49.40 | 20.00 | 1550 | 60/186 | 1919-2006*/  1919-2006* | 9 |
| KRK | Central European, Krk | *Pinus mugo* / Picea abies | Evergreen | 200 | 50.85 | 15.47 | 1400 | 31/101 | 1919-2006*/  1919-2006* | 9 |
| VAG | Central Norwegian Scandes,Vagamo | *Empetrum hermaphroditum* / *Betula pubescens* | Evergreen | 30 | 61.88 | 9.25 | 1450 | 48/14 | 1951-2004/  1951-2004* | 10–11 |
| CS | Northern French Alps, Côte des Salières | *Rhododendron ferrugineum* / *Picea abies* | Evergreen | 80 | 45.05 | 5.85 | 1800- 2100 | 36/16 | 1821-2015/  1800-2015 | 12–13 |
| ZG* | Tibetan Plateau, Zhegu | *Rhododendron przewalskii* / Sabina squamata | Evergreen | 250 | 31.63 | 102.96 | 4050 | 19/45 | 1950-2009/  1950-2009 | 14–15 |
| SYS* | Tibetan Plateau,Sygera-SE | *Rhododendron aganniphum* / Abies georgei | Evergreen | 320 | 29.66 | 94.71 | 4390 | 18/19 | 1950-2012*/  1950-2017* | 16–17 |
| SYN* | Tibetan Plateau,Sygera-N | *Rhododendron aganniphum* / Abies georgei | Evergreen | 280 | 29.64 | 94.70 | 4380 | 17/21 | 1950-2012*/  1950-2017* | 16–17 |
| DL* | Tibetan Plateau, Dulan | *Salix oritrepha* / Juniperus przewalskii | Deciduous | 160 | 36.94 | 98.69 | 3828 | 28/33 | 1960-2015/  1960-1996 | 18,19 |
| WL* | Tibetan Plateau, WL | *Salix oritrepha* / Juniperus przewalskii | Deciduous | 160 | 36.00 | 98.18 | 4270 | 35/71 | 1969-2014/  1950-2004 | 18,20 |

**Table 7.** Final parameter settings used in the VS model.

| **Code** | **Life form** | **T1** | **T2** | **T3** | **T4** | **M1** | **M2** | **M3** | **M4** | **Is** | **M0** | **Mmax** | **Mmin** | **lr** | **Λ** | **Pmax** | **C1** | **C2** | **C3** | **Tsm** | **Cs1** | **Cs2** | **Tbeg** | **Rsm** | **Tsm** |
| --- | --- | --- | --- | --- | --- | --- | --- | --- | --- | --- | --- | --- | --- | --- | --- | --- | --- | --- | --- | --- | --- | --- | --- | --- | --- |
| VOL | Tree | 6.2 | 18.1 | 25.4 | 31.3 | 0.03 | 0.17 | 0.84 | 0.90 | 280 | 0.2 | 0.6 | 0.01 | 1.3 | 0.01 | 29.5 | 0.75 | 0.27 | 0.18 | 27.4 | 11.6 | 0.006 | 77.3 | 1.5 | 0.9 |
|  | Shrub | 6.9 | 20.0 | 23.0 | 31.3 | 0.03 | 0.13 | 0.79 | 0.91 | 354 | 0.1 | 0.6 | 0.06 | 0.9 | 0.02 | 49.2 | 0.80 | 0.27 | 0.18 | 17.4 | 11.4 | 0.005 | 27.2 | 2.5 | 3.0 |
| JES | Tree | 3.8 | 16.1 | 24.6 | 33.1 | 0.05 | 0.19 | 0.79 | 0.92 | 106 | 0.1 | 0.7 | 0.05 | 1.4 | 0.01 | 34.5 | 0.71 | 0.13 | 0.16 | 37.6 | 11.6 | 0.010 | 60.1 | 4.7 | 1.9 |
|  | Shrub | 1.2 | 18.7 | 23.5 | 31.0 | 0.03 | 0.19 | 0.84 | 0.92 | 101 | 0.2 | 0.6 | 0.03 | 0.5 | 0.020 | 28.4 | 0.83 | 0.14 | 0.16 | 18.04 | 8.40 | 0.008 | 42.6 | 2.4 | 2.4 |
| TAT | Tree | 6.5 | 15.4 | 24.5 | 33.7 | 0.05 | 0.10 | 0.81 | 0.88 | 95 | 0.1 | 0.4 | 0.05 | 1.3 | 0.01 | 42.3 | 0.89 | 0.22 | 0.15 | 13.7 | 8.0 | 0.008 | 25.8 | 1.2 | 0.3 |
|  | Shrub | 1.6 | 18.4 | 24.5 | 31.5 | 0.02 | 0.23 | 0.83 | 0.89 | 10 | 0.2 | 0.7 | 0.10 | 1.4 | 0.04 | 20.8 | 0.70 | 0.18 | 0.17 | 10.6 | 9.0 | 0.010 | 83.1 | 5.7 | 1.6 |
| KRK | Tree | 6.4 | 19.5 | 23.5 | 33.1 | 0.04 | 0.17 | 0.84 | 0.92 | 152 | 0.13 | 0.50 | 0.06 | 0.4 | 0.006 | 25.1 | 0.71 | 0.22 | 0.20 | 10.15 | 8.35 | 0.005 | 90.46 | 4.97 | 6.4 |
|  | Shrub | 6.4 | 18.5 | 23.6 | 32.0 | 0.06 | 0.24 | 0.81 | 0.88 | 268 | 0.08 | 0.38 | 0.09 | 0.5 | 0.013 | 49.8 | 0.84 | 0.14 | 0.17 | 10.67 | 11.12 | 0.009 | 96.01 | 4.26 | 6.4 |
| VAG | Tree | 6.3 | 16.6 | 22.2 | 30.3 | 0.02 | 0.25 | 0.79 | 0.94 | 358 | 0.3 | 0.7 | 0.08 | 0.5 | 0.01 | 36.0 | 0.70 | 0.12 | 0.16 | 18.9 | 11.2 | 0.004 | 79.5 | 1.2 | 0.3 |
|  | Shrub | 1.2 | 15.1 | 23.5 | 30.3 | 0.05 | 0.11 | 0.77 | 0.88 | 120 | 0.2 | 0.6 | 0.02 | 0.7 | 0.00 | 38.8 | 0.77 | 0.15 | 0.20 | 3.3 | 11.0 | 0.009 | 26.0 | 5.2 | 0.4 |
| CS | Tree | 4.8 | 17.5 | 24.9 | 32.7 | 0.05 | 0.15 | 0.83 | 0.94 | 17 | 0.2 | 0.4 | 0.09 | 0.6 | 0.04 | 33.4 | 0.75 | 0.26 | 0.16 | 21.0 | 8.8 | 0.004 | 54.1 | 3.4 | 1.7 |
|  | Shrub | 1.7 | 18.1 | 25.4 | 30.5 | 0.03 | 0.19 | 0.80 | 0.86 | 445 | 0.2 | 0.4 | 0.09 | 0.7 | 0.01 | 26.4 | 0.75 | 0.15 | 0.19 | 15.0 | 10.2 | 0.005 | 56.8 | 2.7 | 1.9 |
| ZG | Tree | 5.4 | 19.6 | 24.5 | 31.2 | 0.02 | 0.20 | 0.77 | 0.92 | 21 | 0.1 | 0.6 | 0.10 | 1.0 | 0.01 | 38.4 | 0.72 | 0.16 | 0.20 | 3.6 | 12.0 | 0.006 | 61.1 | 5.3 | 0.6 |
|  | Shrub | 5.1 | 15.3 | 24.5 | 30.9 | 0.03 | 0.14 | 0.83 | 0.91 | 204 | 0.2 | 0.5 | 0.04 | 0.6 | 0.01 | 20.9 | 0.79 | 0.28 | 0.16 | 6.4 | 8.1 | 0.008 | 72.4 | 1.1 | 1.9 |
| SYS | Tree | 1.4 | 17.0 | 25.8 | 31.7 | 0.03 | 0.26 | 0.77 | 0.86 | 290 | 0.3 | 0.7 | 0.06 | 0.4 | 0.04 | 35.0 | 0.90 | 0.10 | 0.18 | 29.0 | 9.3 | 0.005 | 99.7 | 1.7 | 2.1 |
|  | Shrub | 2.0 | 16.8 | 22.1 | 32.7 | 0.04 | 0.20 | 0.77 | 0.92 | 485 | 0.3 | 0.6 | 0.02 | 0.5 | 0.05 | 23.7 | 0.72 | 0.23 | 0.17 | 20.9 | 8.5 | 0.007 | 85.0 | 1.1 | 3.0 |
| SYN | Tree | 1.3 | 17.9 | 22.3 | 32.0 | 0.06 | 0.21 | 0.76 | 0.90 | 270 | 0.1 | 0.5 | 0.10 | 0.4 | 0.01 | 24.5 | 0.95 | 0.20 | 0.19 | 19.1 | 11.9 | 0.007 | 75.8 | 1.8 | 1.1 |
|  | Shrub | 2.6 | 15.3 | 24.0 | 33.2 | 0.06 | 0.13 | 0.80 | 0.87 | 350 | 0.2 | 0.6 | 0.02 | 0.9 | 0.01 | 34.8 | 0.93 | 0.30 | 0.16 | 28.5 | 11.2 | 0.008 | 50.1 | 2.9 | 0.5 |
| DL | Tree | 1.3 | 18.2 | 25.9 | 33.6 | 0.04 | 0.24 | 0.83 | 0.87 | 462 | 0.2 | 0.5 | 0.04 | 1.4 | 0.02 | 48.6 | 0.88 | 0.25 | 0.19 | 37.5 | 12.0 | 0.008 | 80.6 | 2.8 | 2.0 |
|  | Shrub | 2.7 | 18.0 | 23.0 | 30.4 | 0.05 | 0.30 | 0.78 | 0.92 | 193 | 0.3 | 0.4 | 0.02 | 1.2 | 0.05 | 44.2 | 0.83 | 0.17 | 0.19 | 2.9 | 9.1 | 0.008 | 89.9 | 1.2 | 1.1 |
| WL | Tree | 3.6 | 18.1 | 22.6 | 31.7 | 0.05 | 0.17 | 0.77 | 0.86 | 144 | 0.2 | 0.4 | 0.01 | 1.4 | 0.01 | 26.3 | 0.89 | 0.14 | 0.16 | 31.8 | 10.6 | 0.007 | 89.2 | 2.0 | 0.3 |
|  | Shrub | 6.8 | 19.8 | 25.4 | 31.2 | 0.06 | 0.20 | 0.85 | 0.86 | 447 | 0.1 | 0.5 | 0.01 | 0.5 | 0.01 | 47.4 | 0.88 | 0.11 | 0.16 | 8.4 | 9.5 | 0.008 | 76.7 | 1.1 | 1.9 |

T_1_: Minimum temperature for growth [°C];

T_2_: Lower threshold of optimal temperatures [°C];

T_3_: Upper threshold of optimal temperatures [°C];

T_4_: Maximum temperature for growth [°C];

M_1_: Minimum soil moisture for growth [v/v];

M_2_: Lower threshold of optimal soil moisture [v/v];

M_3_: Upper threshold of optimal soil moisture [v/v];

M_4_: Maximum soil moisture for growth [v/v];

I_s_: Initial snowpack (mm);

M_0_: Initial soil moisture [v/v];

M_max_: Field capacity [v/v];

M_min_: Wilting point [v/v];

L_r_: Rooting depth [m];

Λ: Coefficient of water drainage from soil [-];

P_max_: Maximum precipitation for soil saturation [mm];

C_1_: Proportion of precipitation not intercepted by canopy [-];

C_2_: First coefficient of transpiration [mm/day];

C_3_: Second coefficient of transpiration [1/°C];

T_sm_: Heat sum for start soil melting (C);

C_s1_: 1st coefficient of soil melting;

C_s2_: 2nd coefficient of soil melting;

T_beg_: Cumulative temperature threshold to onset growth [°C];

R_sm_: Rate of snow melting (mm/C/day);

T_sm_: Minimum temperature for snow melting.

**Table 8.** Comparison of linear mixed models assessing the effects of spring forcing and different chilling temperature ranges on the early cambial phenology of shrubs and trees at two treelines located on the south-eastern Tibetan Plateau. Models are sorted following the Akaike Information Criterion (AIC), including sites and species as random effects. The best model is highlighted in bold; df, degrees of freedom.

| Models | Shrubs | | Trees | |
| --- | --- | --- | --- | --- |
|  | df | AIC | df | AIC |
| Forcing | 4 | 382.11 | 5 | 743.59 |
| Chilling (0~5 °C) | 4 | 501.04 | 5 | 805.39 |
| Chilling (-5~5 °C) | 4 | 502.23 | 5 | 806.17 |
| Chilling (-5~0°C) | 4 | 499.43 | 5 | 805.47 |
| Chilling (-10~0 °C) | 4 | 500.93 | 5 | 805.06 |
| Forcing+Chilling (0~5 °C) | 5 | 377.98 | 6 | 748.25 |
| Forcing+Chilling (-5~5 °C) | 5 | 354.24 | 6 | 745.99 |
| **Forcing+Chilling (-5~0°C)** | **5** | **351.38** | **6** | **733.97** |
| Forcing+Chilling (-10~0 °C) | 5 | 386.08 | 6 | 736.25 |
| Forcing×Chilling (0~5 °C) | 6 | 388.24 | 7 | 758.90 |
| Forcing×Chilling (-5~5 °C) | 6 | 361.79 | 7 | 753.10 |
| Forcing×Chilling (-5~0°C) | 6 | 357.46 | 7 | 735.58 |
| Forcing×Chilling (-10~0 °C) | 6 | 394.94 | 7 | 746.38 |

**Table 9.** Mean annual temperature and precipitation data used for modeling early cambial phenology at alpine treeline sites located across the Northern Hemisphere. Tmean, Tmax, Tmin and Pre indicate mean, maximum, and minimum temperatures and precipitation, respectively. E-OBS, MEK, LZ, DL, WL indicate daily climate data from the E-OBS 26.0 0.1º-gridded database, Maerkang (MEK), Linzhi (LZ), Dulan (DL) and Wulan (WL) meteorological stations, respectively.

| Site code | Time span | Tmean (ºC) | Tmax (ºC) | Tmin (ºC) | Pre (mm) | Source |
| --- | --- | --- | --- | --- | --- | --- |
| VOL | 1950-2020 | -0.41 | 6.77 | -8.20 | 521.16 | E-OBS |
| JES | 1950-2020 | 1.72 | 8.34 | -4.97 | 947.43 | E-OBS |
| TAT | 1950-2020 | 1.29 | 8.04 | -5.67 | 842.45 | E-OBS |
| KRK | 1950-2020 | 1.04 | 7.46 | -5.56 | 1153.97 | E-OBS |
| VAG | 1950-2020 | -0.89 | 5.87 | -8.45 | 439.79 | E-OBS |
| CS | 1950-2020 | 2.57 | 8.49 | -3.83 | 1149.37 | E-OBS |
| ZG | 1955-2014 | 0.72 | 10.55 | -5.34 | 777.78 | MEK |
| SYS | 1960-2019 | -0.21 | 6.07 | -3.69 | 665.58 | LZ |
| SYN | 1960-2019 | -0.68 | 6.23 | -4.76 | 665.58 | LZ |
| DL | 1964-2017 | 1.77 | 9.67 | -4.79 | 210.79 | DL |
| WL | 1969-2018 | -0.88 | 5.50 | -6.02 | 201.88 | WL |

**Supplementary references**

1. Rossi S, *et al*. Pattern of xylem phenology in conifers of cold ecosystems at the Northern Hemisphere. *Glob Change Biol* 2016; **22**: 3804–3813.
2. Deslauriers A, *et al*. Cambial phenology, wood formation and temperature thresholds in two contrasting years at high altitude in southern Italy. *Tree Physiol* 2008; **28**: 863–871.
3. Gričar J, *et al*. Plastic and locally adapted phenology in cambial seasonality and production of xylem and phloem cells in *Picea abies* from temperate environments. *Tree Physiol* 2014; **34**: 869–881.
4. Li X, *et al*. Warming menaces high-altitude Himalayan birch forests: evidence from cambial phenology and wood anatomy. *Agric For Meteorol* 2021; **308–309**: 108577.
5. Prislan P, *et al*. Precipitation is not limiting for xylem formation dynamics and vessel development in European beech from two temperate forest sites. *Tree Physio.* 2018; **38**: 186–197.
6. Li X, *et al*. Temperature thresholds for the onset of xylogenesis in alpine shrubs on the Tibetan Plateau. *Trees* 2016; **30**: 2091–2099.
7. Weijers S, *et al*. Recent spring warming limits near-treeline deciduous and evergreen alpine dwarf shrub growth. *Ecosphere* 2018; **9**: e02328.
8. Rössler O, Löffler J. Uncertainties of treeline alterations due to climatic change during the past century in the central Norwegian Scandes. *Geoöko* 2007; **28**: 104–114.
9. Šenfeldr M, *et al.* Diverging growth performance of co-occurring trees (*Picea abies*) and shrubs (*Pinus mugo*) at the treeline ecotone of Central European mountain ranges, *Agric For Meteorol* 2021; **108608**: 308–309.
10. Bär A, *et al*. Ring-width chronologies of the alpine dwarf shrub *Empetrum hermaphroditum* from the Norwegian mountains. *IAWA J* 2007; **28**: 325–338.
11. Bär A, *et al*. Growth-ring variations of dwarf shrubs reflect regional climate signals in alpine environments rather than topoclimatic differences. *J Biogeogr* 2008; **35**: 625-636.
12. Francon L, *et al.* Warm summers and moderate winter precipitation boost *Rhododendron ferrugineum* L. growth in the Taillefer massif (French Alps). *Sci Tot Env* 2017; **586**: 1020-1031.
13. Francon L, *et al.* Shrub growth in the Alps diverges from air temperature since the 1990s. *Env Res Lett* 2021; **16**: 074026.
14. Li Z, *et al*. The growth-ring variations of alpine shrub *Rhododendron przewalskii* reflect regional climate signals in the alpine environment of Miyaluo Town in Western Sichuan Province, China. *Acta Ecol Sinica* 2013; **33**: 23–31.
15. Li Z, *et al*. Tree-ring based summer temperature reconstruction over the past 200 years in Miyaluo of western Sichuan, China. *Quat Sci* 2011; **31**: 522-534.
16. Lu X, *et al.* Up to 400-year old Rhododendron shrubs on the southeastern Tibetan Plateau: prospects for shrub-based dendrochronology. *Boreas* 2015; **44**, 760–768.
17. Liu B, *et al*., Topography and age mediate the growth responses of Smith fir to climate warming in the southeastern Tibetan Plateau. *Int J Biometeorol* 2016; **60**, 1577–1587.
18. Lu X, *et al.* Climate response of *Salix oritrepha* growth along a latitudinal gradient on the northeastern Tibetan Plateau. *Dendrobiology* 2019; **81**: 14–21.
19. Fang K, *et al.* Response of regional tree-line forests to climate change: evidence from the northeastern Tibetan Plateau. *Trees* 2009; **23**: 1321–1329.
20. Zhu H, *et al.* Millennial temperature reconstruction based on tree-ring widths of Qilian juniper from Wulan, Qinghai Province, China. *Chin Sci Bull* 2008; **53**: 3914–3920.
21. Bunn AG. A dendrochronology program library in R (dplR). *Dendrochronologia* 2008; **26**: 115–124.
22. R Core Team. R: A language and environment for statistical computing. R Foundation for Statistical Computing, Vienna, Austria. https://www.R-project.org/ (2020).
23. Rossi S, *et al*. Pattern of xylem phenology in conifers of cold ecosystems at the Northern Hemisphere. Glob Change Biol 2016; 22: 3804–3813.
24. Cornes R, *et al*. An ensemble version of the E-OBS temperature and precipitation datasets. *J Geophys Res Atmos* 2018; **123**: 9391-9409.
25. Anchukaitis KJ *et al.* An interpreted language implementation of the Vaganov–Shashkin tree-ring proxy system model. *Dendrochronologia* 2020; **60**: 125677.
26. Vaganov EA *et al.* Growth Dynamics of Conifer Tree Rings: Images of Past and Future Environments. Ecological, Springer-Verlag, Berlin, 2006.
27. Tumajer J *et al.* Growing faster, longer or both? Modelling plastic response of *Juniperus communis* growth phenology to climate change. *Glob Ecol Biogeogr* 2021; **30**: 2229–2244.
28. Buttò V*et al*. Comparing the cell dynamics of tree-ring formation observed in microcores and as predicted by the Vaganov–Shashkin Model. *Front Plant Sci* 2020; **11**: 1268.
29. *Katoch* S *et al.* A review on genetic algorithm: past, present, and future. *Multimed. Tools Appl.* 2021; **80**: 8091–8126.
30. Rossi S *et al*. Critical temperatures for xylogenesis in conifers of cold climates. *Glob Ecol Biogeogr* 2008; **17**: 696–707.
31. Treml V, *et al*. Differences in growth between shrubs and trees: How does the stature of woody plants influence their ability to thrive in cold regions? *Agric For Meteorol* 2019; **271**: 54–63.
32. Huang J, *et al.* Photoperiod and temperature as dominant environmental drivers triggering secondary growth resumption in Northern Hemisphere conifers. *Proc Natl Acad Sci U.S.A.* 2020; **117**: 20645–20652.
33. Ren P, *et al.* Critical temperature and precipitation thresholds for the onset of xylogenesis of *Juniperus przewalskii* in a semi-arid area of the north-eastern Tibetan Plateau. *Ann Bot* 2018; **121**, 617–624.
34. Li X, *et al.* Critical minimum temperature limits xylogenesis and maintains treelines on the southeastern Tibetan Plateau. *Sci Bull* 2017; **62**: 804-812.
35. Wang Y, *et al*. Phenological variation in height growth and needle unfolding of Smith fir along an altitudinal gradient on the southeastern Tibetan Plateau. *Trees* 2013; **27**, 401–407.
36. Zhang J, *et al*. Terminal bud size, spring and summer temperatures regulate the timing of height-growth cessation of Smith fir on the southeastern Tibetan Plateau. *Agric For Meteorol* 2022; **316**: 108883.
37. Liang E, *et al*. Species interactions slow warming-induced upward shifts of treelines on the Tibetan Plateau. *Proc Natl Acad Sci U.S.A.* 2016; **113**, 4380–4385.
38. Wang Y, *et al*. Increased stem density and competition may diminish the positive effects of warming at alpine treeline. *Ecology* 2016; **97**: 1668–1679.
